# Lower 25-hydroxyvitamin D (25OHD) levels, diabetes and age are associated with foot and ankle fracture treatment complications

**DOI:** 10.20945/2359-4292-2022-0020

**Published:** 2023-09-22

**Authors:** Hallan Douglas Bertelli, José Luis Braga de Aquino, Vânia Aparecida Leandro-Merhi

**Affiliations:** 1 Pontifícia Universidade Católica de Campinas Escola de Ciências da Vida Campinas SP Brasil Pontifícia Universidade Católica de Campinas (PUC-Campinas), Escola de Ciências da Vida, Programa de Pós-graduação em Ciências da Saúde, Campinas, SP, Brasil

**Keywords:** 25-hydroxyvitamin D (25OHD), albumin, treatment, complications, nutritional status, foot and ankle fractures

## Abstract

**Objective:**

This study investigated the nutritional status, 25-hydroxyvitamin D (25OHD), albumin and risk factors associated with complications in patients with foot and ankle fragility fractures.

**Subjects and methods:**

Prospective study, developed with patients who suffered fractures due to fragility of the foot and ankle (n = 108); the type of fractured bone, fracture mechanisms and classification were studied and also pseudoarthrosis, treatment, surgical dehiscence, anthropometry, 25OHD and albumin. The Chi-square or Fisher's exact test, Mann-Whitney and Kruskal-Wallis tests were used in the statistical analysis and the multiple logistic regression analysis was used to identify the risk factors associated with complications.

**Results:**

The factors that, together, were associated with treatment complications were the level of 25OHD (p = 0.0055; OR = 0.868 [1,152]; 95% CI = 0.786; 0.959 [1.043;1.272]) and diabetes (p = 0.0034; OR = 30,181; 95% CI = 3.087; 295.036). The factors that, together, were associated with the presence of any complication, were age (p = 0.0139; OR = 1.058; 95% CI = 1.011; 1,106) and 25OHD level (p = 0.0198; OR = 0.917; 95% CI = 0.852; 0.986). There was a complication probability above 0.40 associated with lower 25OHD levels (values below 20 ng/mL) and older age (over 50 years).

**Conclusion:**

Lower or abnormal levels of 25OHD were associated with pseudoarthrosis, and age and 25OHD were both risk factors for treatment complications in patients with foot and ankle fractures.

## INTRODUCTION

Bone is a dynamic tissue which structure and integrity changes according to lifestyle, diet, physical exercise, sun exposure, among other factors (1). Currently, different studies have investigated the increase of orthopedic fractures incidence and the risk factors associated to those fractures, such as body mass index (BMI), nutritional status, as well as diseases linked to such factors, including osteoporosis, obesity, malnutrition, hypovitaminosis, diabetes and cardiovascular diseases (2).

Vitamin D helps to maintain calcium and phosphorus levels in the body, contributing to proper bone mineralization, making bone tissue less susceptible to problems such as osteoporosis and fractures (3).

Low vitamin D levels are particularly prevalent in patients with orthopedic trauma (4). A study with 899 trauma patients found that 77% had an insufficient vitamin D general rate (≤30 ng/mL) and 39% had a deficiency of such vitamin (≤20 ng/mL) (4). Another study assessed 617 patients who suffered fractures in general and it was observed that 40% of these patients had a vitamin D deficiency, with 11% suffering a severe deficiency (5). In a study conducted with 75 patients with foot and ankle fractures, the percentage of patients with vitamin D deficiency was 47%, with 11% of them presenting with severe deficiency (6).

Another relevant factor regarding low levels of vitamin D is their association with delayed fracture healing and elective arthrodesis. Brinker and cols. (2008) (7), found that two thirds of patients with unexplained pseudoarthrosis after fractures had hypovitaminosis. Moore and cols. (2017) (8), found that vitamin D deficiency or insufficiency was associated with an 8.1-fold increase in pseudoarthrosis after elective foot and ankle surgery. In another retrospective study of 37 military recruits with stress fractures, an association was demonstrated between the time required for recovery and the level of vitamin D; those with levels > 30 ng/mL recovered in significantly less time than those with lower levels (9).

Finally, serum albumin concentration in conjunction with vitamin D deficiency may be related to post-fracture and postoperative complications, such as pseudoarthrosis and infections in orthopedic patients (10–13).

Some scholars have identified the hip as the most susceptible fracture site associated with vitamin D deficiency and hyperparathyroidism (14). However, the impact of changes in bone metabolism, in the bone microstructure and in the type of fracture associated with vitamin D deficiency remains an open question (14).

In light of the above, the purpose of this study was to investigate the nutritional status, 25-hydroxyvitamin D (25OHD), albumin and risk factors associated with complications in patients with fractures due to foot and ankle fragility.

## SUBJECTS AND METHODS

### Study cases, type and characteristics, inclusion and exclusion criteria

In this investigation, the population studied consisted of 108 adult patients diagnosed with ankle and foot bone fractures, attended in a hospital emergency room and monitored for 30 days in an orthopedics outpatient clinic of a large hospital in the State of São Paulo, Brazil. This is a prospective study, carried out between February 2019 and December 2020, after approval by the institution's Ethics and Research Committee and the patients entering the free and informed consent form (FICF) (Opinion No. 3.740.745, CAAE: 21465819.9.0000.5481).

We adopted as inclusion criteria in the study, individuals of both genders, aged over 18 years, diagnosed with a fractured foot and/or ankle bone, victims of low energy trauma or stress fractures detected by foot and ankle simple radiography; low energy trauma was defined as falls from standing height, direct gait pattern trauma, twisting, dancing or sports injury or repetitive strain injury.

Cases of patients under the age of 18 years, with open physeal plaque, who were undergoing 25OHD replacement therapy, victims of high energy traumas (car accidents or falls from heights greater than 1.5 meters), and polyfractured individuals were excluded from the study, as well as individuals who did not accept to participate in the study, who, therefore did not sign the consent form.

### Methodological procedures

Initially, patients were evaluated in an orthopedics outpatient clinic and underwent a medical history and orthopedic physical examination. The diagnosis of foot or ankle fracture was raised and was confirmed through simple radiographs taken at the study site. After confirmation of fractures, patients underwent laboratory and nutritional status evaluations. The following variables were assessed: age, gender, presence of diabetes mellitus, menopause, smoking, bone which was fractured, fracture mechanism, fracture classification, pseudoarthrosis, type of treatment proposed (*surgical or conservative*), surgical dehiscence, weight, height, body mass index (BMI), calf circumference (CC), 25OHD and albumin serum levels.

#### Anthropometric indicators evaluation

Anthropometric parameters of body weight, height and CC were measured, according to standardized procedures and instruments. BMI was calculated based on weight and height measurements. BMI was classified according to the cutoff points that have been established by the World Health Organization (2000) (15), for individuals up to 65 years of age (*underweight, normal weight, overweight and obesity*). And for elderly individuals, over 65 years of age, BMI was assessed according to the cutoff points proposed by the Pan American Health Organization (PAHO-2002) (16) (*low weight, normal weight, overweight and obesity*). For the CC classification, the criteria recommended by the European Consensus on Sacopenia (2010) (17) were taken into account to identify patients with loss of muscle mass (*<34 cm for men and <33 for women*).

#### Albumin and vitamin D laboratory evaluation

The albumin and 25OHD biochemical parameters were assessed at the time of the fracture diagnosis that is before the beginning of the clinical treatment or in the preoperative of surgical cases. In the analysis of laboratory exams and their interpretation, internationally recognized and validated parameters were used. For serum albumin, the following classification was adopted (18): severe depletion: <2.4 mg/dL; moderate depletion: 2.4-2.9 mg/dL; mild depletion: 3.0-3.5 mg/dL and normal: >3.5 mg/dL.

For 25OHD evaluation, the cutoff points recommended by the Brazilian Society of Endocrinology (19–21) and also the values updated by the Brazilian Society of Endocrinology (22) were used. The findings were expressed as “vitamin D status” (*based on thresholds for categorizing patients as being deficient or not*) and also as 25OHD concentration.

The 25OHD analyzes were performed in the Hospital's external laboratory, coordinated by Grupo Fleury's laboratory medicine. The laboratory assay method used the chemiluminescent competitive immunoassay technology for the quantitative determination of 25OHD.

#### Surgical characteristics

Patients who received surgical treatment underwent specific surgical techniques according to the affected region and type of fracture, according to their classifications, regarding the use of absolute stability methods with interfragmentary compression or relative stability and regarding the use of an internal or external tutor (23). The implants used were 2.7 mm or 3.5 mm plates, and 2.7 mm, 3.5 mm or 4.5 mm spongy cortical and locking screws, blocking fibula rods, double compression cannulated screws and Kirshner wires.

#### Post-treatment evaluation and complications

The clinical and surgical results and the complications resulting from the treatments proposed to each patient were evaluated two and four weeks after the diagnosis, and the following complications were reviewed: wound dehiscence or infection that required surgical intervention; episodes of deep venous thrombosis (DVT) with or without pulmonary thromboembolism (PTE); need to remove synthesis material (23); pseudoarthrosis or no bone consolidation requiring a change in the therapeutic option or surgical revision (23).

#### Statistical analysis

For the statistical analysis, the Chi-square test or Fisher's exact test was used; when necessary, to compare proportions. To compare continuous measurements between two groups, the Mann-Whitney test was applied and, among three groups, the Kruskal-Wallis test. Later, to identify the risk factors associated with complications, multiple logistic regression analysis was used. The stepwise variable selection process was employed (24–26). The level of significance adopted for the statistical tests was 5%.

## RESULTS

The average age of the population studied (n = 108) was 50.5 ± 15.9 years, composed of 69.4% (n = 75) female and 30.6% (n = 33) male patients. In the anthropometric indicators, the average body weight was 75.8 ± 13.1 kg; height 166.6 ± 9.4 cm; BMI 27.3 ± 4.0 kg/m^2^; and the CC was 34.6 ± 2.6 cm. In the laboratory tests, the mean serum albumin was 4.2 ± 0.4 mg/dL and 25OHD was 26.2 ± 8.6 ng/mL.

As for the fractured bone, the ankle was affected in 51.9% (n = 56) of the cases; a total of 61.5% (n = 32) of the ankle fractures were classified as unimalleolar, 21.2% (n = 11) as bimalleolar and 17.3% (n = 9) as trimalleolar. Fall was the most frequent injury mechanism, occurring in 50.9% (n = 55) of the cases; direct traumas caused 20.4% (n = 22) of the fractures, sprains were responsible for 16.7% (n = 18) of the injuries, stress fractures affected 9.3% (n = 10) of the cases and the practice of football injured 2.8% (n = 3) of the patients. As for the treatment proposed to the 108 patients who participated in the investigation, conservative treatment was elected for 50.9% (n = 55) of the patients and surgical treatment for 49.1% (n = 53) (Table 1).

**Table 1:** Distribution of the population according to gender, habits, comorbidities and treatment

Variables	Classification	%	N
Gender	Female	69.4	75
	Male	30.6	33
Fractured Bone	Foot	48.1	52
	Ankle	51.9	56
Fractured Bone	2nd Metatarsus	12.0	13
	5th Metatarsus	10.2	11
	Calcaneus	76.5	7
	Finger	13.0	14
	Hallux	3.7	4
	Tibial pestle	3.7	4
	Talus	2.8	3
	Ankle	48.1	52
Ankle Fractures	Unimalleolar	61.5	32
	Bimalleolar	21.2	11
	Trimalleolar	17.3	9
Fracture Side	Bilateral	1.9	2
	Right	58.3	63
	Left	39.8	43
Mechanism	Sprain	16.7	18
	Stress	9.3	10
	Football	2.8	3
	Fall	50.9	55
	Trauma	20.4	22
Treatment	Surgical	49.1	53
	Conservative	50.9	55
Smoking	No	91.7	99
	Yes	8.3	9
Diabetic	No	85.2	92
	Yes	14.8	16
Menopause	No	42.7	32
	Yes	57.3	43

The majority of patients, 87.0% (n = 94), had not suffer any complications within 30 days of the proposed treatment. However, 6.5% (n = 7) evolved with pseudoarthrosis, 1.9% (n = 2) with pseudoarthrosis and deep vein thrombosis (DVT), 3.7% (n = 4) of the cases with dehiscence and only 0.9% (n = 1) of the cases presented dehiscence of the surgical wound and required removal of the synthesis material.

Table 2 shows the population distribution according to anthropometric indicators and laboratory tests.

**Table 2:** Population distribution according to anthropometric measurements, laboratory tests and 25-hydroxyvitamin D (25OHD) concentration (n = 108)

Variables		%	N
Body mass index			
	Low weight	0.9	1
	Normal	32.4	35
	Overweight	47.2	51
	Obesity	19.4	21
Calf circumference			
	Loss of muscle mass	22.2	24
	No loss	77.8	84
Albumin			
	Depletion	4.6	5
	Normal	95.4	103
Vitamin D status*			
	Normal	32.4	35
	Deficiency	24.1	26
	Insufficiency	43.5	47
25-hydroxyvitamin D**			
	≤30 (ng/mL)	67.6	73
	>30 (ng/mL)	32.4	35
25-hydroxyvitamin D***			
	<20 (ng/mL)	24.1	26
	≥20 (ng/mL)	75.9	82

**25-hydroxyvitamin D**	**Mean ± SD**	**Median (min-max)**	**N**
(ng/mL)	26.2 ± 8.6	27.0 (6.0-55.0)	108

* Source: Brazilian Society of Endocrinology [Maeda and cols., 2014 (19)] and Holick and cols., 2011 (20).

** Brazilian Society of Endocrinology [Maeda and cols., 2014 (19)] and Holick and cols., 2011(20), unifying deficiency and insufficiency.

*** Brazilian Society of Endocrinology [(Ferreira and cols., 2018 (22)].

When analyzing the variables assessed (*age, gender, body weight, height, BMI, CC,* 25OHD*, albumin, type of treatment [surgical or conservative], smoking, comorbidities, complications*) and their associations with foot and ankle fractures only albumin (p = 0.0016), type of treatment (p < 0.0001) and postoperative complications (p = 0.0157), showed a statistically significant difference. Regarding the type of bone affected, there was an association only with the type of treatment (p < 0.001). On the other hand, in the relationship between all variables and the type of treatment (surgical or conservative), there was an association with the weight of the patients (p = 0.0177) and with the CC (p = 0.0191); patients who underwent conservative treatment had lower weight and CC measurements compared to those who underwent surgical treatment. Regarding gender (p = 0.0153), fractured bone (p < 0.001) and complications (p = 0.0468) occurred in a greater proportion in women, in patients with foot fractures and pseudoarthrosis and in patients undergoing conservative treatment, respectively. Although the other variables did not present significant differences in relation to the type of treatment, it was observed that 75.5% of patients undergoing surgical treatment and 60% of the patients undergoing conservative treatment were overweight. Regarding 25OHD, normal values were observed only in 26.4% of patients undergoing surgical treatment and in 38.2% of the patients undergoing conservative treatment.

In the assessment of variables and associations with complications, two analyses were carried out, the first one separating complications in three different ways, “*dehiscence*”, “*pseudoarthrosis*” and “*no complications”*, with significant values in relation to the patient's age (p = 0.0367). The patients who developed dehiscence were older (67 ± 17.0 years), compared to those who had no complications. As to the fractured bone, there was a higher proportion of foot fractures that evolved into pseudoarthrosis (p = 0.0157). There were even more complications associated with patients with a history of diabetes (p = 0.0024) and a higher proportion of dehiscence in ankle fractures in these patients. The other variables did not show significant differences in relation to complications.

In the investigation of the risk factors associated with complications, through multiple logistic regression analysis, generalized logit model and stepwise variable selection process, the factors that jointly were associated with complications were the level of 25OHD (p = 0.0055; OR = 0.868 (1.152); 95% CI = 0.786; 0.959 (1.043;1.272)) and diabetes (p = 0.0034; OR = 30,181; 95% CI = 3.087; 295.036). Each unit less of 25OHD increased 15% the chance of patients developing pseudoarthrosis and the presence of diabetes increased 30-fold the chance of dehiscence (Table 3).

**Table 3:** Study of risk factors associated with complications, assessed by multiple regression analysis

Variable	Reference	Complication	Value p	OR	95% CI
25-hydroxyvitamin D (25OHD)	Numeric	Dehiscence x none	0.9362	0.995	0.877; 1.129
25-hydroxyvitamin D (25OHD)		Pseudoarthrosis x none	0.0055	0.868 (1.152)	0.786; 0.959 (1.043;1.272)
Diabetic	Yes x No	Dehiscence x none	0.0034	30.181	3.087; 295.036
Diabetic	Yes x No	Pseudoarthrosis x none	0.9832	0.976	0.100; 9.564

OR: odds ratio; 95% CI: 95% confidence interval for OR.

The other variables (gender, body weight, height, BMI, CC, albumin, bone type, smoking and menopause) were tested, but showed no significant association. The 25-hydroxyvitamin D (25OHD) concentration was associated with poor outcomes after adjusting for diabetes.

When the complications were split into “*no complications*” and “*with complications*”, an association was found with age (p = 0.0178), and it was observed that older patients (60.57 ± 13.10 years) experienced more complications. There was also a higher proportion of diabetic patients with post-fracture complications (p = 0.0333), and menopausal patients experienced a higher proportion of complications in foot and ankle bone fractures (p = 0.0371). The other variables evaluated did not present a significant association with the complications, when classified as “*no complications*” and “*with complications*”.

Table 4 shows that, according to the multiple logistic regression analysis, the factors that, together, were associated with the presence of “any complication”, were age (*p=0.0139; OR=1,058; 95% CI=1,011; 1,106*) and 25OHD level (*p=0.0198; OR=0.917; 95% CI=0.852; 0.986*). Each additional year of age is associated with an increased chance of complication by 5.8%; and each unit less of 25OHD increased 9.1% the chances of complications.

**Table 4:** Factors associated with any complication, analyzed by multiple logistic regression

Variable	Reference	p-value	OR	95% CI
Age	Numeric	0.0139	1.058	1.011; 1.106
25-hydroxyvitamin D (25OHD)	Numeric	0.0198	0.917(1.091)	0.852; 0.986(1.014; 1.173)

OR: odds ratio; 95% CI: 95% confidence interval for OR.

The other variables (gender, body weight, height, BMI, CC, albumin, bone type, smoking and menopause) were tested, but showed no significant association. The 25-hydroxyvitamin D (25OHD) concentration was associated with poor outcomes after adjusting for age.

The control variables are listed in the Tables 3 and 4. The other variables (gender, body weight, height, BMI, CC, albumin, bone type, smoking and menopause) were tested but resulted in no significant association. The 25OHD concentration was associated with poor outcomes after adjusting the variables for age (Table 4) and diabetes (Table 3). It was described in the multiple model by the stepwise selection process. The inverse of the Odds Ratio shows that those are low values of 25OHD, which change the chances of poor outcomes (Tables 3 and 4).

**Figure 1:**
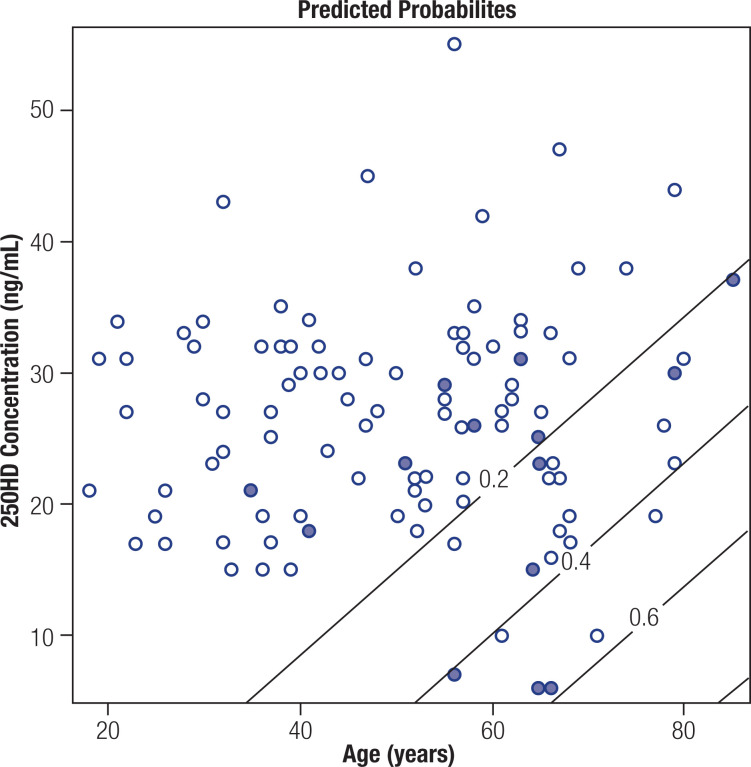
Probabilities estimated by logistic regression for the presence of complications, according to the variables selected in the stepwise multiple model process. Probability above 0.40 was related to lower 25-hydroxyvitamin D (25OHD) values (*values less than 20*) and older age (*age over 50*). The slanted lines in the graph represent the cutoff values in the estimated probabilities. The darkest dots represent patients with an estimated probability of complication above 0.6.

Figure 1 illustrates the probabilities of complications estimated by logistic regression, according to the variables selected in the multiple model, with a stepwise process. There was a probability of complications above 0.40 associated with lower 25OHD levels (*values less than 20*) and older age (*over 50*). It was observed that as age increases and serum 25OHD levels decrease, the likelihood of complications increases.

The slanted lines in the graph represent the cutoff values in the estimated probabilities. The darkest dots represent patients with an estimated probability of complication above 0.6.

It was also verified through the Spearman correlation coefficient that there was no significant correlation between the concentration of 25OHD and albumin (coefficient = 0.1684; p = 0.0814).

## DISCUSSION

This study showed that in patients with fractures due to foot and ankle fragility, after 30 days follow-up, there was a greater tendency for conservative treatments in female and slimmer patients, with lower weight and lower calf circumference; greater tendency for dehiscence in foot and ankle surgical cases in older patients with diabetes mellitus; greater tendency for ankle fractures due to fall mechanism, with a greater proportion of muscle mass loss (reduced calf circumference) in patients who suffered trimalleolar fractures; greater tendency for pseudoarthrosis in patients undergoing conservative treatment after foot fractures.

This investigation's findings showed that complications in foot and ankle fractures are not uncommon, and the factors related to these complications must be analyzed and corrected beforehand to reduce injury to patients. Schmidt and cols. (2020) (27) found that about 9.1% of patients undergoing foot and ankle surgical procedures presented complications such as arthrodesis, amputations and debridement. This study's data showed that older patients (mean 67 ± 17.0 years) had a greater tendency to dehiscence in foot and ankle fractures surgeries. Koval and cols. (2005) (28) and Srinivasan and cols. (2001) (29) also observed that age was a factor associated with complications after foot or ankle fractures.

In the regression analysis, reviewing the complications separately, it was observed that each unit less of 25OHD, increased 15% the chances of the patient presenting pseudoarthrosis. And diabetes increased 30-fold the chances of dehiscence. When we reviewed the presence or absence of complications jointly, the outcome found was that each additional year in the individual's age increased by 5.8% the chances of the patient developing some type of complication, and with each unit less in serum 25OHD these chances increased by 9.1%. Our investigation also pointed out a probability above 0.40 of lower 25OHD levels, as noted in Figure 1.

Other studies reviewed the relationship of 25OHD serum levels and complications, such as pseudoarthrosis; Donnally and cols. (2019) (30) conducted a retrospective study with 150 patients after lumbar arthrodesis and found that 25OHD values were not significantly associated with the rates of postoperative pseudoarthrosis, the need for surgical revision and surgical complications. Kerezoudis and cols. (2016) (31) using a systematic review concluded that patients who will undergo a lumbar arthrodesis procedure, can benefit from the correction of 25OHD deficiency to reduce the risks of surgical complications such as pseudoarthrosis and the need for revision. Brinker and cols. (2008) (7) assessed 683 patients with bone pseudoarthrosis and selected, using judicious methods, 37 patients, who were examined for metabolic and endocrine abnormalities, as a potential risk factor for pseudoarthrosis, and found that the most common abnormality was 25OHD deficiency, present in 68% of the patients. Moore and cols. (2017) (8) in a case-control study that included 58 patients undergoing foot and ankle elective arthrodesis, sought to identify endocrine and metabolic abnormalities that could be associated with the presence of pseudoarthrosis in those surgeries. The authors (8) split the sample into two 29 patients groups; one group with pseudoarthrosis and the other with bone consolidation, and concluded that patients with 25OHD deficiency or insufficiency were 8.1 more likely to develop pseudoarthrosis (*95% confidence interval, 1,1996 to 32,787)*, the other variables such as age, gender, smoking, BMI or type of procedure were not statistically significant.

Richards and Wright (2020) (9) in a retrospective study with 37 military personnel with stress fractures, analyzed the relationship of serum 25OHD levels and the time needed for fractured bone consolidation and concluded that patients with sufficient serum 25OHD concentration (50 nmol/L) experienced shorter average recovery time.

Another recent study (27) pointed out that patients with diabetes as a previous comorbidity also experienced more complications after fractures (*26.0% of diabetic patients versus 14.6% of non-diabetics, p=0.001*). Wukich and cols. (2010) (32), assessed 1000 patients after foot and ankle surgery and found a higher proportion of infection in diabetics (13.2%) compared to non-diabetics (2.8%). Deep infections were also more common in diabetics (*6.9% versus 1.3%, p=0.001*), who also underwent more unplanned procedures, including debridement, arthrodesis and amputations (*18.3% versus 9.1 %, p=0.001*). Wukich and cols. (2014) (33); in a level 1 evidence study with a large cohort of 2,060 patients who underwent foot and ankle surgery, it was shown that surgical site infections are independently associated with peripheral neuropathy and HbA1C > 8%. Another retrospective study, carried out by Romero and cols. (2020) (34), indicated that, after ankle fractures, 36.5% of patients experienced complications. In other words, complications can occur after fractures and tend to be more common in patients with predisposing factors, such as diabetes.

Finally, the findings of this investigation highlight the existence of an intimate relationship between the occurrence of orthopedic fractures and the control of food intake. Adequate dietary guidance, maintenance of optimal body weight, regular physical activity, sun exposure at non-critical times, diabetes control and adequate levels of 25OHD are suggested in patients who have suffered fractures.

### Study limitations

The limitations of this study refer, first, to the fact that it was carried out in a single location, with a population sample with access to health insurance plans, belonging to a better social class and with the tendency to exhibit higher body mass indexes. Besides, this study did not have control groups composed of individuals without fractures that would allow collection and review of similar type data. Thus, the study authors suggest further investigations in connection with this subject in future studies.

In conclusion lower or abnormal levels of 25OHD were associated with pseudoarthrosis, and age and 25OHD were both risk factors for treatment complications in patients with foot and ankle fractures.

## Data Availability

the datasets supporting the conclusions of this article are available from the corresponding author on reasonable request.
